# Tracking urban activity growth globally with big location data

**DOI:** 10.1098/rsos.150688

**Published:** 2016-04-27

**Authors:** Matthew L. Daggitt, Anastasios Noulas, Blake Shaw, Cecilia Mascolo

**Affiliations:** 1 Computer Laboratory, University of Cambridge, Cambridge, UK; 2 Data Science Institute, Lancaster University, Lancaster, UK; 3 Facebook Inc., Palo Alto, CA, USA

**Keywords:** big location data, social networks, urban growth, urban mobility

## Abstract

In recent decades, the world has experienced rates of urban growth unparalleled in any other period of history and this growth is shaping the environment in which an increasing proportion of us live. In this paper, we use a longitudinal dataset from Foursquare, a location-based social network, to analyse urban growth across 100 major cities worldwide. Initially, we explore how urban growth differs in cities across the world. We show that there exists a strong spatial correlation, with nearby pairs of cities more likely to share similar growth profiles than remote pairs of cities. Subsequently, we investigate how growth varies inside cities and demonstrate that, given the existing local density of places, higher-than-expected growth is highly localized while lower-than-expected growth is more diffuse. Finally, we attempt to use the dataset to characterize competition between new and existing venues. By defining a measure based on the change in throughput of a venue before and after the opening of a new nearby venue, we demonstrate which venue types have a positive effect on venues of the same type and which have a negative effect. For example, our analysis confirms the hypothesis that there is large degree of competition between bookstores, in the sense that existing bookstores normally experience a notable drop in footfall after a new bookstore opens nearby. Other place types, such as museums, are shown to have a cooperative effect and their presence fosters higher traffic volumes to nearby places of the same type.

## 1. Introduction

Urban growth has been a focal point for global development in recent decades. Large cities are often characterized as the steam engines of the world’s economy and understanding the changes our urban environments are undergoing today will be invaluable for guiding and improving the impact of future growth. Fresh approaches to studying urban growth are being aided by the increasing availability of new layers of data describing human activity in urban settings [1]. The analysis and modelling solutions typically proposed in this context have focused on human mobility and transport modelling [2], neighbourhood detection [3,4] and urban scaling [5,6] among others.

However, advances in city science have not only come from work primarily concerned with classical urban geography and ecology problems, but have also been driven by computer science and related mobile technologies that lie at the centre of today’s digital revolution. Traditionally, urban growth patterns and activities have been tracked using means such as census data, real-estate records, and traffic and business surveys. However mapping and sensing technologies, alongside mobile crowdsourcing applications, services and systems have enabled a powerful feedback loop between data-driven solutions and the problems studied in the urban domain.

Location-based services, in particular, have played a vital role, with Foursquare being one of the best examples of this new paradigm. Foursquare is powered by more than 60 million users who crowdsource information about places, neighbourhoods and cities as they move. More than 80 000 applications rely on, and contribute to, its data ecosystem through its API. Six years after its launch, it provides a large-scale global source of location data that describes real-world places and human mobility in urban environments.

In this paper, we build on the legacy of Foursquare location data to provide new perspectives on urban activity growth patterns in cities. Working on a longitudinal multi-city snapshot of the data, we are interested in profiling cities based on their activity growth patterns. We first develop a methodology to track urban growth patterns via new place creation in cities. Next, we propose a technique that exploits dynamic location data to detect areas in a city which are experiencing surges in development. Finally, we investigate how the emergence of new urban activities in a neighbourhood influences existing locations in terms of pedestrian traffic. In detail, we make the following contributions:

— Initially, we provide an intercity perspective on urban activity growth by representing cities as vectors of the frequency of new place types within them. By applying spectral clustering to these vectors, we demonstrate a strong correlation between urban growth patterns and location. In particular, nearby pairs of cities, such as those that belong to the same country or continent, which are far more likely to share a similar urban activity profile than remote pairs of cities. We hypothesize that these observations are a result of archetypal patterns of urban growth shared on a regional level and possibly rooted in similar cultures. In fact, when we represent cities in terms of relative growth places (frequency of new place types versus frequency of existing place types), a method that aims to capture more recent policies affecting urban growth, we show that the spatial correlation in the clustering results is lost.
— Next, we take a more local view on the urban growth process, performing a vertical analysis on each city. Specifically, we analyse the spatial distribution of new places across the city, tracking *where* new places tend to be created. While the influence of a strong urban hierarchy is prevalent, with more new places being created in what is typically known as the urban core of a city, there are examples where accelerated growth in urban development occurs in peripheral areas. Frequently, this phenomenon is due to the existence of large development projects in response to preparation for large events such as the Olympic Games or the World Cup, as we demonstrate with representative case studies in London, UK and Brasília, Brazil.
— Finally, we look at the impact of urban development on existing places. Exploiting user mobility information, we measure how the opening of a new venue can have an effect on local establishments in terms of pedestrian traffic. We identify the formation of two important trends: firstly, the existence of *cooperative* place types that enable larger mobility flows to nearby venues, and secondly, the existence of *competitive* place types whose presence in an area disrupts existing traffic flows to nearby places. Interestingly, the former class of place types includes categories such as monuments, train stations or public spaces that represent anchors of generative urban development, whereas the latter category involves local businesses such as restaurants, pharmacies or barbershops that typically compete for customer traffic. There are exceptions, however, a notable one being the presence of Turkish restaurants, which we discover tend to form local ecosystems that reinforce traffic volumes to other venues of the same type.

Overall, our analysis shows how modern datasets, generated by mobile users as they naturally explore an urban environment, can form the basis for sustainable monitoring frameworks and tools that could be deployed to manage tomorrow’s cities.

## 2. The dataset

The basis of our analysis is a 4-year-long dataset from Foursquare describing movements between places in 100 cities from around the globe. For each Foursquare venue in a city, the dataset contains

— unique ID,
— latitude and longitude,
— creation time,
— general Foursquare category (e.g. *nightlife spot*),
— specific Foursquare category (e.g. *bar*, *jazz club*),
— total number of *check-ins*, and
— total number of unique visitors.

One of the criteria for a venue to have been included in the dataset is that it must have at least 100 total check-ins. While unfortunately removing a few genuine, but very unpopular, venues from the dataset, this filter has the advantage that it removes the vast majority of false venues added either mistakenly or maliciously. To provide a sense of the scale for the venue data, the smallest city contains over 2000 venues, while the largest contains approximately 60 000 venues.

Alongside the venue data, the dataset also contains all *transitions* which occurred within the city in the four-year time period. A transition is defined to be a pair of check-ins by a single user to two different venues less than 3 h apart in time. For each transition, we have

— start time,
— end time,
— source venue ID, and
— destination venue ID.

The transition records contain no information about the identity of the user.

Critically, we have information on the creation time of a venue (i.e. the time that the place was added to the Foursquare database) which should allow us to find newly opened places. However, as Foursquare was only launched in 2009, most places will not in fact be newly opened when first added as a venue in the database. In order to filter out genuinely new places, we calculate a temporal cut-off point per city, before which we assume all venues added were pre-existing. Appendix A contains a full and more detailed description of our filtering methodology.

## 3. Macro-scale analysis

### 3.1. City growth profiles

In this section, we demonstrate that data crowdsourced from location-based services can be used to identify cities and regions where particular urban activities are currently experiencing strong growth.

We should note that we are using the term *growth* somewhat loosely in this context to mean the number of new venues opening up. A more satisfactory way to measure growth would be to look at the change in the number of venues (venues opening minus venues closing). However, the limitations of the dataset mean that we have no reliable way of detecting closing venues.

To begin with, we investigate clustering the cities according to their growth profile in terms of the number of new venues opening within the time period covered by the dataset. The main questions we address are: *Can we use crowdsourced data about places to profile cities in terms of urban activity growth?* And moreover, *How do these profiles of urban growth compare and what is the role of geography in this relationship?*

These questions naturally arise from the observation that cities are famous for their particular composition of place types. For example, many buildings in Cambridge, UK, are directly or indirectly related to the local university and Paris is well known for its many cafés. In this work, we put forward a methodology where the focus is not just on the overall compositions of place types in cities, but how their composition is currently changing as new places are being created. While the nature of such change is interesting in and of itself, as it can shed light on historic and cultural aspects of urban growth, it can also be viewed as a snapshot of current investment in the city. This can highlight the priorities of local government and public spending (*colleges and universities*, *schools*, *government buildings,* etc.) as well as where growth in the private sector is currently focused (*hotels*, *food*, *offices,* etc.).

Before we begin our analysis, we should also note the possibility of heterogeneous Foursquare representation across different categories. For example, it may be that the average Foursquare user is more interested in coffee shops than bowling alleys and, therefore, the former might be overrepresented in the data when compared with the latter. The extent of this potential bias could also be affected by such factors as geography and culture. Given the relatively large size of the Foursquare user base and the length of time the dataset spans, we hope that the effect of this bias is relatively small. Nonetheless, we cannot discount it entirely, and the reader should keep it in mind as a possibility.

#### 3.1.1. Activity growth vectors

For each city, we generate a growth vector **v** whose *i*th component, **v**_*i*_, represents the proportion of new venues in general category *i* as calculated in the following equation:

3.1 $${\text{v}}_{i}=\frac{N_{\mathrm{new}}(i)}{\sum_{i}N_{\mathrm{new}}(i)}.$$

The value *N*_new_(*i*) is the number of new places in the city in category *i*. The categories are from Foursquare’s official list of top-level categories at the time the dataset was created.

**Figure 1.**
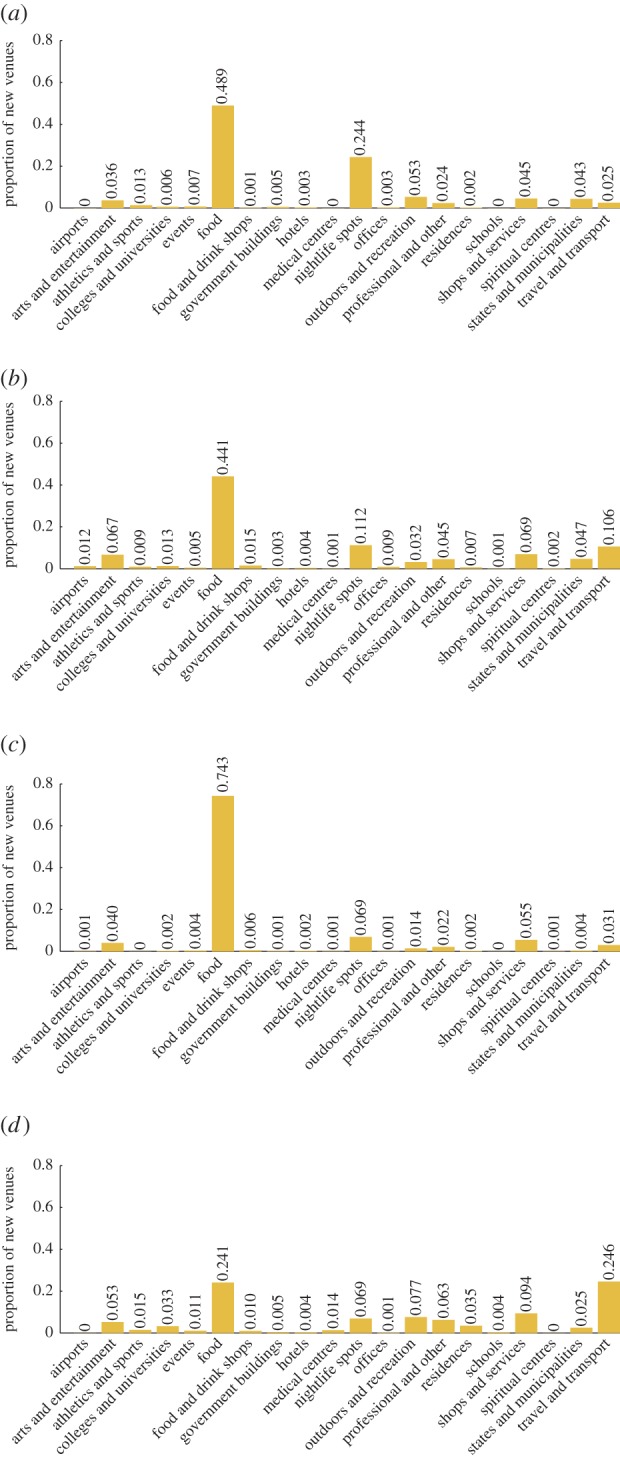
Activity growth profiles for four different cities. (*a*) Athens, (*b*) London, (*c*) Seoul and (*d*) Riga.

As an example, the growth vectors for Seoul, Athens, Riga and London are shown in figure 1. The *food* category is shown to contribute a significant proportion of all new place growth, ranging from 24% in Riga to 74% in Seoul. This is to be expected due to the entrepreneurial nature of food establishments. Accordingly, the average lifespan of *food* venues is relatively low, and so the category undergoes significant churn. Although many features of the growth profiles are shared between cities, such as the prominence of *food* venues, we can also observe differences in the dynamics of urban activity profiles of cities. London’s and Riga’s *travel and transport* categories display relatively high growth, suggesting greater investment in transport infrastructure. Athens *nightlife* category can also be seen to be growing rapidly compared with other categories and cities, amidst the Greek financial crisis.

As a brief aside, we should point out the analysis above does not take the sizes of the new venues into account, an indisputably important factor when measuring growth. One possible alternative analysis might measure the growth in a category by the number of check-ins to the new venues within that category, rather than the number of venues itself. This would be worthy of further study.

#### 3.1.2. Clustering methodology

Louf & Barthelemy [7] apply a methodology for clustering cities based on the topology of street networks and the geometry of the land patches that emerge among the networks of urban streets. They find groups of cities with similar patterns and trace these observations to different urban planning and design strategies adopted by urban authorities. Here, we use a different layer of information that describes a city, specifically the place types or urban activities that emerge as new places open. We cluster cities based on the urban activity growth profiles described in the previous paragraph. Unlike Louf & Barthelemy, we use spectral [7], rather than hierarchical, clustering, as it does not require an initial assumption about the number of clusters [8]. Other similar work includes that of Silva *et al.* [9] who also apply a methodology based on spectral clustering on Foursquare data to measure cultural similarities between cities in terms of *food* venues and culinary preferences.

#### 3.1.3. Results

**Figure 2.**
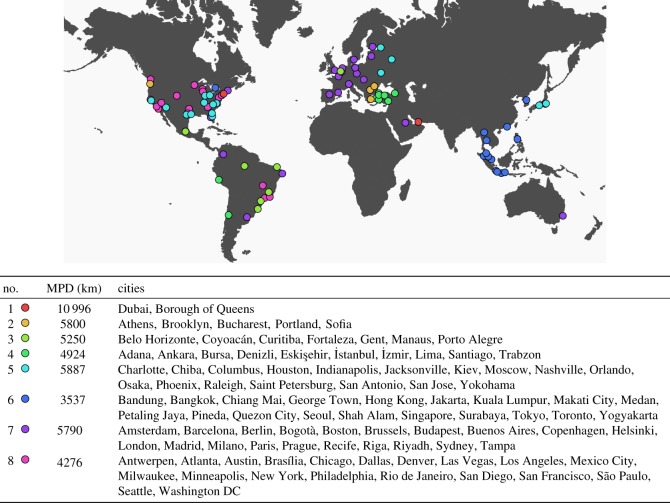
Growth vector clustering results. Mean pair distance (MPD) is the average distance between members of the cluster.

**Figure 3.**
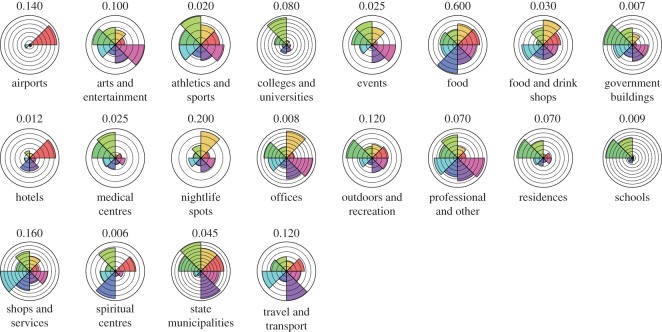
General category components of each cluster. Each graph shows one component of the vectors in figure 1, averaged over the members of the clusters in figure 2. The number on the top of each subfigure is the proportion of the vector that the outermost ring represents (e.g. 13% of all new places in the average country in Cluster 1 belong to the *airports* category).

Figure 2 shows the seven resulting clusters while figure 3 compares the averaged components of each cluster. Notably, there is a clear intra-cluster spatial correlation, suggesting that geography and, by extension, culture play a significant role in determining the urban activity growth of different cities. For example, cluster 2 is predominantly Asian cities, cluster 3 European cities and cluster 8 US cities. As figure 3 shows, in Asian cities *spiritual centres* and *food* places are common urban development activities, whereas US cities focus on *professional and other*, as well as *arts and entertainment* and *sport* activities. European cities, on the other hand, appear to invest in *travel and transport* and *state municipalities*.

While outliers do exist in the composition of the clusters described, those are to some extent expected given that there is some degree of diversity among cities that belong to the same country. For example, US cities split primarily into two clusters which, nonetheless, feature common tics when compared with other clusters, as seen in figure 3. Interestingly, many of the Brazilian cities belong to a cluster whose primary feature is activities related to higher education reflecting recent policies [10] in the country. The same cluster also scores highly for development in *athletics and sports*, which we hypothesize is related to the 2014 World Cup, a major sports event hosted in Brazil. Finally, Dubai and Queens stand out as hubs for *airports* due to the presence of Dubai’s International Airport (the world’s busiest) and LaGuardia Airport that was recently reconstructed.

#### 3.1.4. The role of distance

In order to quantify the spatial correlation, we calculated the mean Haversine distance between pairs of cities in the same cluster. We then randomized the members of each cluster and repeated the calculation. The randomization process fixed the number of clusters and their sizes. Cities were reassigned by first generating a list of all cities from the concatenation of the clusters in a fixed order. This list was then randomly permuted, and the new members of each cluster were chosen by repartitioning the list in the same shape as the original clusters. The results in table 1 show that the mean intra-cluster pair distance is just over half of the corresponding figure if the cities were randomly assigned to clusters without any consideration to geography.

**Table 1.** Quantifying the spatial correlation for clustering by growth vectors.

clustering method	mean distance between intra-cluster pairs (km)
growth vector	4868
random	8752

Given that the magnitude of the *food* component of the city growth vectors dominates other categories (recall figure 1), we recalculated the results using *relative* activity growth vectors. The relative growth vector represents the growth in the category compared with the existing number of venues in that category, rather than the absolute growth. This can be defined as

3.2 $${\text{v}}_{i}=\frac{N_{\mathrm{new}}(i)}{N_{\mathrm{old}}(i)\sum_{j}(N_{\mathrm{new}}(j)/N_{\mathrm{old}}(j))}.$$

For example, although *food* previously carried a significant proportion of the original vector’s weight, it only accounts for a small proportion of the relative growth vector as there are a large number of existing *food* places. One potential concern is that if category *i* is very small then *N*_new_(*i*) divided by *N*_*old*_(*i*) might exhibit somewhat random behaviour. However, we hope that as there are between 2000 and 60 000 venues in each city, in most cases, this is unlikely to have a significant effect.

When clustering was performed using this new measure, although the algorithm returned approximately the same number of clusters and the clusters were of comparable size, the spatial correlation within the clusters largely disappeared. Table 2 shows that the mean distance between intra-cluster pairs was little different than if they had been randomly assigned to clusters.

**Table 2.** Quantifying the spatial correlation for clustering by relative growth vectors.

clustering method	mean distance between intra-cluster pairs (km)
relative growth vector	7716
random	8297

We hypothesize the existing composition of place types in a city is a result of strong cultural and geographical influences. The relative growth vector, by definition, emphasizes more recent trends in urban growth. It thus captures the short-term evolution of urban activities beyond the standard historical and regional patterns. It is, therefore, more likely to reflect local organizational factors such as municipal policies or short-duration infrastructure projects. This type of growth, as indeed table 2 suggests, may be unlikely to have as strong a geographical correlation.

### 3.2. Detecting surges in urban growth

So far, we have provided a cross city, global overview of urban activity growth patterns. We have seen that cities which belong to the same country or continent are more likely to feature similar urban activity profiles. However, the data available through location-based services feature very high spatial granularity and today the position of real-world places is known with accuracy down to 5 m or less [11]. This provides an opportunity to study urban activity growth patterns at an intra-city level. Motivated by these observations, in this section, we ask: *Can data from location-based services be exploited to identify areas in cities where there is a surge in urban development?*

**Figure 4.**
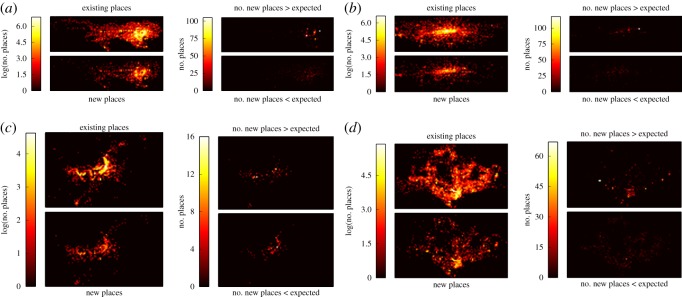
Intensity plots of new places in cities. Upper left: log(existing places); lower left: log(new places); upper right: number of new places less than expected; and lower right: number of new places greater than expected. (*a*) Tokyo, (*b*) London, (*c*) Brasília and (*d*) Singapore.

To answer this, we first investigate the spatial distribution of new places within cities. Initially we divide up each city into a grid with 10 000 identically sized cells and measure the urban activity per cell. We create spatial intensity plots for the distribution of both existing venues and new venues, examples of which can be seen on the left-hand side of figure 4. Having observed that the spatial distribution of new venues does not always coincide with that of existing venues, we now present a methodology for comparing the difference between them.

Our null model randomly redistributes new venues so that the density of new venues in each cell is proportional to the density of the cell’s existing venues

3.3 $$n_{i,j}^{\mathrm{null}}=\frac{n_{i,j}^{\mathrm{existing}}n^{\mathrm{new}}}{n^{\mathrm{existing}}},$$

where $n_{i,j}^{\mathrm{null}}$ represents the number of new venues in cell (*i*,*j*), and *n*^new^ and *n*^existing^ are the total number of new and existing venues in the city, respectively. By subtracting the true distribution of new venues from that of the null model (equation (3.4)), we highlight where new place growth is higher and lower than expected:

3.4 $$v_{i,j}=n_{i,j}^{\mathrm{null}}-n_{i,j}^{\mathrm{new}}.$$

Figure 4 shows the results for four example cities. The most obvious trend is that areas of higher than expected levels of new places are highly concentrated, as opposed to areas of lower than expected development which are scattered broadly. This pattern suggests that development happens in a coordinated fashion, whereas deprivation in development is not planned. Two possible explanations for this coordination could be that it is either directly dictated by the city’s authorities (for example, urban rejuvenation projects), or be a result of a more organic process of emerging development hotspots reinforcing the incentives for further places opening locally.

Interestingly, it is also possible to pick out from these plots several notable developments and events. In London, our methodology is able to detect the development of the the Olympic Village built for the 2012 Olympic Games in Stratford (white dot). Another example is Brasília where one can spot the Mané Garrincha National Stadium rebuilt for the 2014 Football World Cup (rightmost yellow dot). There examples demonstrate how the digital datasets emerging from location-based technologies can become an important tool for monitoring urban development on large scale and with unprecedented geographical accuracy.

## 4. Micro-scale analysis

We have looked at how the emergence of new urban activities in cities can be tracked and showed how the urban development profiles result in clusters of cities that are geographically and culturally proximate. Further, we have demonstrated a method that allows for the detection of surge in urban development across a city’s territory. In other words, we have explored the effects of new place creation in cities as a whole.

We now turn our investigation to how a new venue can influence traffic between other venues in the local area. Answering this question is of interest for the sake of understanding the impact of investment on certain urban activities on a large scale. It has also implications for important problems in retail geography [12–14], such as deciding where to open a new business in a city.

A critical question to ask in this setting is whether a new venue cooperates with nearby venues by increasing footfall in the area, or instead competes, with customers being redirected from existing venues to the new venue’s premises. While various theories have been proposed on the subject since the 1920s [15], new data available from location-based services offer the opportunity to empirically investigate these phenomena on a large scale. In particular, we ask *How does the opening of different venue categories affect pedestrian traffic to nearby venues?*

Our core assumption is that the actual popularity of a venue, as measured by the number of visitors, is correlated with the number of check-ins by mobile users. Of course, a venue’s popularity is a complicated function of many variables including, but not limited to consumer awareness, one-off events and even the state of the country’s economy. It is therefore important to note that when measuring the change in traffic to an existing venue after a new venue opens up, we do not imply a causal effect. However, the aggregation of a sufficiently large number of new and existing venue pairs and mobility data records should allow us to discern overall trends and obtain informative signals on the interaction and reciprocal influence of urban activities in neighbourhoods.

*Definining an ‘impact’ metric*. To do so, we now define a metric to measure this change in users, which we will call ‘impact’. A new venue’s impact on an existing venue is defined to be the ratio between the normalized average number of transitions per month involving the existing venue in the six-month time period before and after the new venue is added to the database. As described in §2, a transition is a pair of check-ins by a single user to two different venues within 3 h of each other.

Formally, the impact metric is defined as

4.1 $$\text{impact}(e,m)=\frac{\sum_{d=m}^{m+5}(t_{e}(d,d+1)/n_{e}(d,d+1))}{\sum_{d=m}^{m-5}(t_{e}(d-1,d)/n_{e}(d-1,d))},$$

where *e* is the existing venue, *m* is the month when the new venue was added to the database, *t*_*e*_(*x*,*y*) is the number of transitions involving *e* that took place between dates *x* and *y* and *n*_*e*_(*x*,*y*) is a normalization constant for venue *v* between dates *x* and *y*. The normalization constant *n*_*v*_(*x*,*y*) takes into account the overall change in monthly Foursquare usage on a city-by-city basis. It also normalizes with respect to the monthly transition counts for all venues in the city of the same specific category. This should remove both seasonal factors (e.g. *ice cream parlours*) and categories undergoing dramatic changes in popularity.

For example an impact value of 1.28 represents a 28% increase in the average normalized number of transitions per month after the new venue opens, while 0.92 represents an 8% decrease. We also remove from our analysis any new–old venue pairs that have less than six months of transition data, either before or after the opening of the venue. Finally, the six-month average was chosen as a compromise value. Given that the dataset covers a time period of 4 years, too long a measurement period would remove a sizeable portion of the dataset from consideration, while too short a period would result in significant noise being added to the impact values.

### 4.1. Homogeneous urban activity interactions

We now explore the effects of new places opening up in close proximity to existing places that are of the same category. That is, they represent the same urban activity. As an initial study, it has the advantage that one would expect minimal differences between the times venues of the same type are active. Naturally, if venues do not share similar opening times, they are unlikely to interact directly. A nightclub and a primary school, even if they are on the same street, are unlikely to exchange customer flows or disrupt each other. As a result, this approach removes the need to account for temporal variability in venue usage.

In this analysis, we take advantage of the depth of detail present in the Foursquare dataset and consider specific, rather than general, Foursquare place types. Whereas in the previous analysis we viewed, for example, *Italian restaurants* and *Japanese restaurants* as part of the *food* category,^1^ we now consider them as distinct categories in their own right.

#### 4.1.1. Case studies: burger joints, bookstores and airport gates

As an initial case study, we investigate the *burger joints*, *bookstores* and *airport gates* categories in London. We chose this particular subset of categories as *burger joints* is the fastest-growing specific category in London, the *bookstores* category is one we hypothesize to be naturally competitive and *airport gates* should be naturally cooperative (as new gates are indicative of expansion in the airport capacity). Using KD-trees, an efficient spatial look-up algorithm [16], we identified all instances of a new venue opening up within 500 m of an existing venue of the same specific category. For each pair, we then calculated the impact of the new venue on the existing venue as defined in equation (4.1).

**Figure 5.**
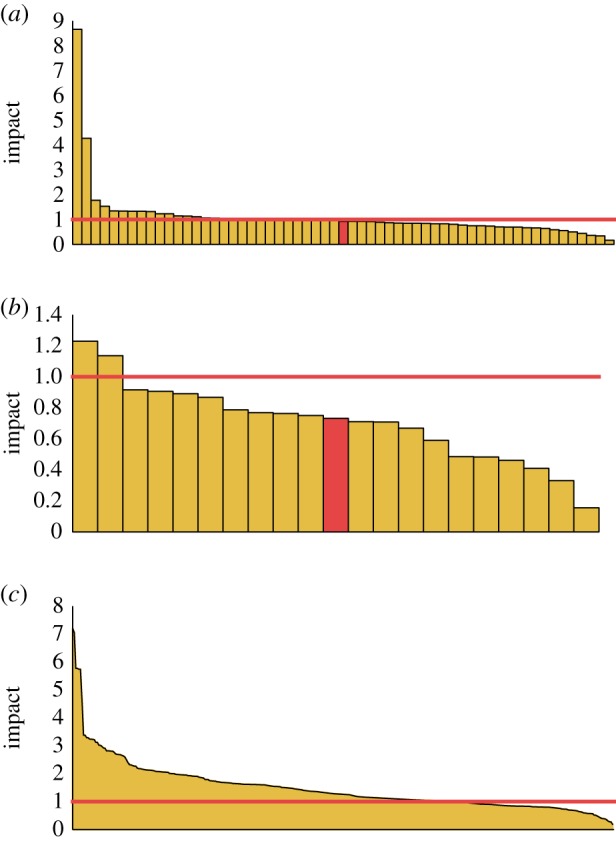
Impact values of all new–existing pairs in London. (*a*) Burger joints (59 pairs), (*b*) bookstores (21 pairs) and (*c*) airport gates (352 pairs). Each bar represents a new venue opening up within 500 m of an existing venue. An impact value of greater than 1.0 indicates a positive effect on the existing place type, less than 1.0 a negative impact. The red bar indicates the median pair.

The results for the three categories are shown in figure 5. The mean change in average monthly transitions for an existing burger joint after a new burger joint opened up within 500 m is an increase of 8.5%. At first glance, this suggests that burger joints may have a positive effect on footfall to the area. However, this mean increase is the result of two outliers. The median change is in fact a decrease of 12.4%. This suggests that new burger joints in fact steal customers from existing burger joints. As hypothesized, 19 out of the 21 existing bookstores experienced a significant decrease in traffic after another bookstore opened up nearby. Similarly, the majority of airport gates experience an increase in traffic after a new airport gate opens up nearby.

#### 4.1.2. Top competitive and cooperative categories

Given the encouraging results from the case studies, we performed similar analysis for all categories in the dataset that had at least 10 new–old pairs. Given the high variance observed in figure 5, in the following analysis, we rank the categories by both the median and the mean impact. This is because the mean can often be misleading as a measure of typical behaviour in distributions with high variance.

Tables 3 and 4 show the top 10 categories that have the lowest median and mean impact, respectively. These can be characterized as the categories that are the most competitive. Food-related categories make up a significant percentage of the most competitive places, with the *pharmacies* and *bookstores* categories also being present.

**Table 3.** Top competitive categories by median impact.

no.	category	median
1	coworking spaces	0.415
2	tapas restaurants	0.632
3	grocery stores	0.636
4	cosmetics shops	0.666
5	train stations	0.673
6	pharmacies	0.673
7	salad shops	0.681
8	sandwich places	0.720
9	salons/barbershops	0.724
10	offices	0.725

**Table 4.** Top competitive categories by mean impact.

no.	category	mean
1	grocery stores	0.678
2	bookstores	0.702
3	pharmacies	0.727
4	seafood restaurants	0.753
5	Asian restaurants	0.761
6	ice cream shops	0.813
7	capitol buildings	0.825
8	salons/barbershops	0.834
9	tapas restaurants	0.838
10	gastropubs	0.842

Tables 5 and 6 show the top 10 categories that have the highest median and mean impact, respectively. These can be characterized as the categories that are the most cooperative. Many of the entries are to be expected. When a new museum opens next to an existing museum it increases the likelihood of the desirability of the area. It is also common for them to run local promotions with joint tickets for other nearby museums. Likewise, a new academic building opening is likely to be part of the same educational establishment of nearby academic buildings and hence likely foreshadows an expansion in the capacity of that establishment. We also note that there is a high degree of agreement between the median and the mean, with 7 of the 10 entries being shared between both lists.

**Table 5.** Top cooperative categories by median impact.

no.	category	median
1	neighbourhoods	1.480
2	Turkish restaurants	1.354
3	gardens	1.341
4	monuments	1.337
5	plazas	1.285
6	tea rooms	1.264
7	airport gates	1.260
8	churches	1.182
9	museums	1.115
10	bus stations	1.106

**Table 6.** Top cooperative places by mean impact.

no.	category	mean
1	museums	3.236
2	roads	1.902
3	convention centres	1.890
4	plazas	1.736
5	academic buildings	1.543
6	airport gates	1.492
7	neighbourhoods	1.489
8	bus stations	1.467
9	tea rooms	1.380
10	Turkish restaurants	1.365

The oddity in the list of cooperative places is the *Turkish restaurant* category. Restaurants (and subcategories of *food* in general) tend to be competitive as table 7 shows. After all, a customer is unlikely to eat twice, no matter how many restaurants are in an area. We explore this anomaly further in the next section.

**Table 7.** Restaurant categories ranked by median impact.

restaurant type	median impact	mean impact
tapas	0.632	0.838
Asian	0.731	0.761
seafood	0.735	0.753
Mediterranean	0.807	1.298
Chinese	0.811	0.868
Indian	0.827	0.868
Vietnamese	0.844	0.901
Japanese	0.874	0.886
sushi	0.886	0.939
Mexican	0.908	0.895
vegetarian/vegan	0.925	0.874
Korean	0.929	0.998
Thai	0.945	1.023
French	0.974	0.957
American	0.997	1.012
Italian	1.007	1.065
Middle Eastern	1.115	1.233
Turkish	1.354	1.365

#### 4.1.3. Explaining cooperation between Turkish restaurants

In the previous section, we showed that Turkish restaurants showed a surprising degree of cooperation, with existing Turkish restaurants experiencing a median increase in customers of 35.4% in the six months after a new Turkish restaurant opened nearby. A potential social hypothesis for this is that the Turkish community forms highly concentrated enclaves in London. Under this hypothesis, ethnic Turkish shops and restaurants open up in existing Turkish communities and create an ecosystem effect, further increasing the likelihood of new Turks settling in the area.

If this hypothesis holds then one would expect very tight clustering of Turkish restaurants in limited geographical areas. To test this, we turn to Jensen’s quality metric [14], which measures whether the local density of the type of venues is greater or lesser than expected if the spatial distribution of place types was a product of a random process. The following equation defines his intra-coefficients for places of the same type:

4.2 $$M_{A}=\frac{N_{t}-1}{N_{A}(N_{A}-1)}\sum_{i=1}^{N_{A}}\frac{N_{A}(A_{i},r)}{N_{t}(A_{i},r)},$$

where *N*_*t*_ is the total number of places, *N*_*A*_ is the number of places of type *A*, *N*_*t*_(*A*_*i*_,*r*) is the total number of places within a radius *r* of the *i*th place of type *A* and *N*_*X*_(*A*_*i*_,*r*) is the number of places of type *X* within a radius *r* of the *i*th place of type *A*.

**Figure 6.**
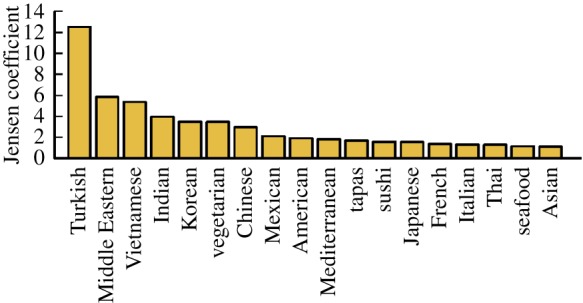
Jensen quality for various restaurant types in London.

Figure 6 plots Jensen’s intra-coefficients for each specific restaurant sub-category using a radius of 500 m. The results show that Turkish restaurants are on average 12 times more densely clustered than would be expected if they were distributed at random. This is far higher than the other restaurant types, providing further evidence for our hypothesis that an enclave effect in the Turkish community is affecting the placement of new Turkish restaurants.

## 5. Discussion and concluding remarks

In our work, we have exploited data sourced from location-based services to reveal urban growth patterns. Tracking these patterns on a global scale, as our cross-city clustering analysis has shown, can reveal interesting trends in urban development where the role of geography and culture remains important, even in an era where centralized city planning is dominant.

However, as our intra-city analysis has shown and confirming recent proposals [17], the power of big location data could allow the detection of large on-going projects across the urban territory. This is an indicator of the potential of developing modelling frameworks and software tools that could allow the monitoring of such projects and thus enable more effective city governance. The study of the interactions of urban activities as measured by Foursquare place categories and foot traffic not only provides further evidence for the advantages of this approach, but also provides the opportunity to study the impact of urban growth on nearby places and, in particular, retail establishments. Previous research has suggested [12,14] that the growth and success of the latter category appear to be closely related to the formation of the neighbouring urban ecosystem.

Overall, our findings complement the current research activities in urban data science. New layers of data are brought together to understand better urban processes and city life. From user survey data that help us improve our understanding of urban spaces [18,19], to exploiting user mobility dynamics and street network patterns [7,20–24] in order to build new models and tools describing cities, the prospects for the smart cities of the future look promising.

## Figures and Tables

**Figure 7.**
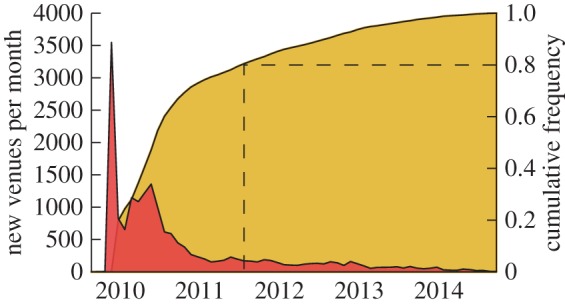
Venues by added per month. The dotted line represents our cut-off point between existing and new venues.

**Figure 8.**
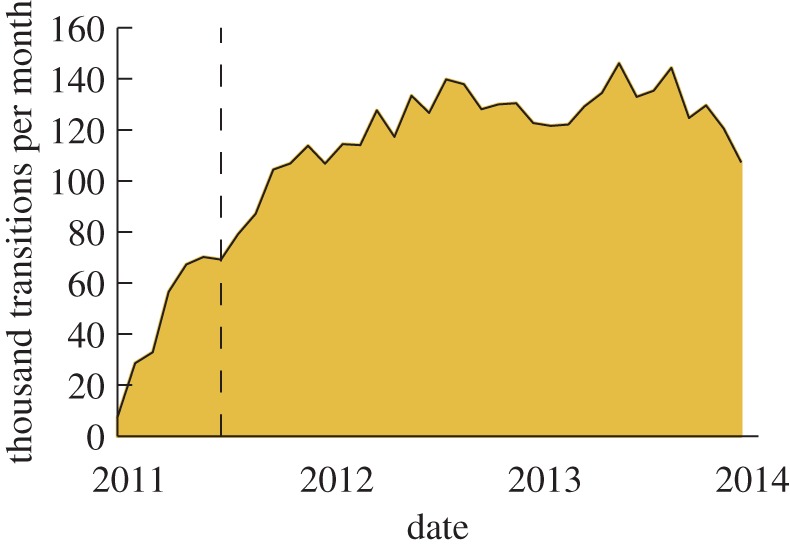
Foursquare’s changing popularity as measured by transitions per month. The dotted line represents our cut-off point between existing and new venues.

## Footnotes

1 See https://developer.foursquare.com/categorytree.

## Data accessibility

The Foursquare dataset used in the present work is proprietary and cannot be directly shared by the authors without permission from Foursquare. However, the majority of the venue data used in §3 can be obtained from the public Foursquare API. Unfortunately, due to changes to the API, the transition data used in §4 can no longer be publicly accessed via the API at the time of publishing. In case of interest, readers are welcome to contact the authors to discuss approaching Foursquare for access. Alternatively, similar datasets can be accessed through the OpenStreetMap project where crowdsourced data about points-of-interest are available.

## Appendix A. Identification of new places

This paper analyses the impact of new places in cities. However, a precursor to any such analysis is the ability to identify which Foursquare venues are new places. As Foursquare was launched in 2009 with an empty database of venues, for any given venue the exact meaning of the *date added* field is unknown. It may represent the approximate date the place opened, or it may be that the place pre-dates Foursquare and it represents the first visit of a Foursquare user.

To help overcome this we assume that, at some point in time, Foursquare adoption was sufficiently large that only a small fraction of venues remain unvisited by Foursquare users. Figure 7 shows the rate of new places being added to the London dataset over time and figure 8 shows how the number of transitions in London varies over time.

As expected, a large proportion of all venues were added to the dataset at the beginning of the period studied. Under our hypothesis, these are existing places being added. Subsequently, the number of new venues added decays sharply. After studying data from the other cities in the dataset, we conclude that the stated hypothesis seems reasonable. We found empirically that across nearly all cities the last 20% of venues added to the database are contained in the comparatively flat tail of the distribution. Therefore, we take the date where the number of new venues reaches 80% of the city’s total to be the cut-off point between new and existing venues. This point in time, assuming it exists, will be city-dependent.

Unfortunately, the rate of new venues added does still gradually decrease after these cut-off points. Lacking any credible evidence that the growth rate of new places in cities is decreasing worldwide, we are forced to accept that we are still capturing some residual existing venues among the genuinely new venues. Unfortunately, we have to accept this as a limitation of the dataset. We argue that the majority of venues we capture are indeed new venues as:

— Figure 8 shows that Foursquare usage remains roughly constant in London from 2012 onwards. Therefore, the venues added after the cut-off date are not simply unpopular existing venues found by a rapidly growing user-base.
— As mentioned in §2, in order to have been included in the dataset, a venue must have had at least 100 check-ins. The greater a venue’s total check-ins, the greater the probability of it being a genuinely new venue, as the likelihood of it not having been previously visited by a Foursquare user is increasingly remote. For example, in order for an existing venue to be added in 2013, no Foursquare user could ever have checked-in beforehand, but over 100 users subsequently checked-in from 2013 to 2014. While not impossible, this does not seem likely to occur frequently.
— Finally, in order to provide a rough estimate of our accuracy, we selected 50 random venues from our list of new venues in London. For each of these, we attempted to manually ascertain whether they were genuinely new places.

As can be seen in table 8, only four places (less than 10% of the sample) turned out to be existing places and two of these were a restaurant and a hotel that had closed for several years to undergo lengthy refurbishment and therefore could be argued to be new in the context of our analysis looking at the effects of places opening up. Of 14 unverified places, 13 were chain stores (Starbucks, River Island, etc.) for which it is very difficult to find the precise opening date of an individual store. Given the turn-over of such chain stores, it is easy to believe that the majority of them were indeed new.

**Table 8.** Manual verification of 50 ‘new places’.

genuinely new	unverifiable	existing places
32	14	4
